# Association between triglyceride glucose-body mass index and incident risk of heart failure among patients with type 2 diabetes: a real-world study

**DOI:** 10.3389/fendo.2026.1798721

**Published:** 2026-05-07

**Authors:** Zhe Zhang, Baoze Qu, Qiuning Wang

**Affiliations:** 1 Department of Cardiology, The First Affiliated Hospital of Jinzhou Medical University, Jinzhou, China; 2 School of Basic Medical Sciences, Jinzhou Medical University, Jinzhou, China

**Keywords:** heart failure, insulin resistance, real world study, triglyceride glucose-body mass index, type 2 diabetes

## Abstract

**Objective:**

The triglyceride glucose-body mass index (TyG-BMI) is recognized as a reliable indicator for assessing insulin resistance. The association between TyG-BMI and heart failure (HF) has been less studied, with inconsistent findings. We aimed to investigate the relationship between TyG-BMI and HF in among the Chinese populations with type 2 diabetes.

**Methods:**

This retrospective cohort study included 4116 patients with type 2 diabetes, comprising 2941 men and 1175 women. TyG-BMI levels were determined utilizing the equation. The primary outcome was the diagnosis of HF, confirmed by cardiologists utilizing ICD-10-CM code I50. Cox proportional hazards models were utilized to evaluate the relationship between TyG-BMI and HF. Receiver operating characteristic (ROC) curve analysis was performed to evaluate the discriminative ability of TyG-BMI, BMI, and the TyG index. Shapley Additive Explanations (SHAP) analysis was applied to interpret the contribution of each variable within the prediction model. Additionally, the Sprague-Dawley rats were used to illustrate alterations in glucose and lipid metabolic parameters in the context of impaired cardiac function.

**Results:**

Over a median follow-up of 3.01 years, 904 new cases of HF were identified. After adjusting for multiple factors, the hazard ratios (HRs) [95% confidence interval (CI)] of HF based on quartiles of TyG-BMI levels at baseline [<175 (set as the reference), 175-208, 209-250, ≥ 250] were 1.00, 1.11(95% CI 0.89-1.45), 1.17 (95% CI 0.94-1.45), and 1.40 (95% CI 1.13-1.73), respectively. The TyG-BMI index exhibited robust predictive capability for incident HF, with an area under the curve (AUC) of 0.83, in contrast to 0.80 for BMI and 0.72 for the TyG index. Rats with impaired cardiac function exhibited elevated levels of blood glucose and lipids, as evidenced by animal research.

**Conclusion:**

In the overall population with type 2 diabetes, a higher TyG-BMI is associated with increased risk of HF. In comparison to TyG and BMI, the TyG-BMI index exhibits superior predictive value for incident HF.

## Introduction

Heart failure (HF) is a clinical syndrome characterized by a range of symptoms and signs, arising from a multitude of pathophysiological factors. Predominantly caused by various cardiovascular diseases, HF represents a significant public health concern and ranks among the foremost causes of mortality (1). The analysis of data from the Global Burden of Disease (GBD) 2021 study revealed a substantial rise in the global prevalence of HF, as well as the burden of disability-adjusted life years and the age-standardized prevalence from 1990 to 2021 (2). Moreover, forecasts suggest that the population of individuals with HF in the United States would increase to 8.7 million by 2030, 10.3 million by 2040, and 11.4 million by 2050 (3). Although advancements in HF treatment strategies have improved patients’ quality of life, mortality and re-hospitalization rates remain elevated. Consequently, the enhancement of early screening and intervention for individuals at high risk of HF has emerged as a critical strategy to mitigate morbidity and mortality.

Recent research has established a notable correlation between insulin resistance (IR) and HF regarding their etiology and pathogenesis, suggesting that these conditions mutually influence and exacerbate one another (4–6). The hyperinsulinemic-euglycemic clamp technique is acknowledged as the gold standard for evaluating insulin sensitivity (7); however, its invasive characteristics and time-consuming procedure render it unfeasible for extensive clinical research. The triglyceride glucose-body mass index (TyG-BMI) has recently been proposed as a straightforward metric for assessing IR. The TyG-BMI index combines three parameters: triglycerides (TG), fasting plasma glucose (FPG), and body mass index (BMI), resulting in a composite measure that reflects lipid metabolism, glucose metabolism, and the extent of obesity. The formula for calculating TyG-BMI is: [ln (fasting TG (mg/dL) × FPG (mg/dL)/2)] × BMI (kg/m^2^) (8). Emerging clinical evidence suggests TyG-BMI is linked to an increased risk of diabetes, hypertension, non-alcoholic fatty liver disease, and cardiovascular diseases (9–12).

However, there is a scarcity of studies investigating the relationship between TyG-BMI and the incidence of HF in the Chinese population. The aim of this study was to investigate the correlation between TyG-BMI and the incidence of HF. Additionally, the Sprague-Dawley rats were used to illustrate alterations in glucose and lipid metabolic parameters in the context of impaired cardiac function.

## Methods

### Study participants

The study population consisted of patients with type 2 diabetes for whom anthropometric, demographic, laboratory, diagnostic, and prescription data were available. Clinical data were retrieved from the electronic medical records (EMRs) of the First Affiliated Hospital of Jinzhou Medical School covering the period from January 1, 2021, to December 31, 2024. The study protocol was reviewed and approved by the Institutional Review Board of the First Affiliated Hospital of Jinzhou Medical University (Approval No. 250154-5). As the analyzes were based on de-identified EMRs, the requirement for informed consent from individual participants was waived.

**Figure 1:**
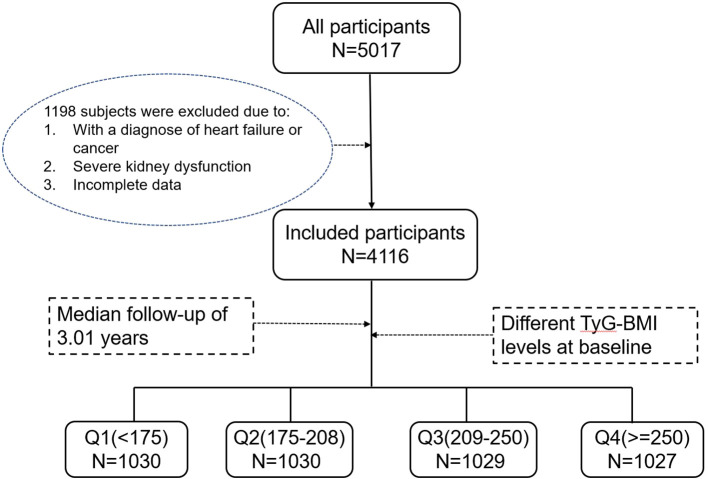
Flowchart of participant selection.

Patients with type 2 diabetes who had at least two regular visits annually at the First Affiliated Hospital of Jinzhou Medical School were eligible for this study. The baseline date was selected as the date of their first physician visit with available data. A total of 1,198 patients were excluded from the analysis due to either diagnosis of heart failure or cancer at baseline (n = 420), severe kidney dysfunction (n = 395), or incomplete data (n = 383). Finally, a total of 4,116 patients were finally included, as shown in **Figure 1**.

### Measurements

Information regarding patients’ date of birth, gender, smoking status, and pharmaceutical prescriptions—including antihypertensive, glucose-lowering, lipid-lowering, and antiplatelet or anticoagulant medications—was acquired from the EMRs. The BMI index was determined by dividing weight in kilograms by the square of height in meters. Furthermore, patients’ blood pressure, serum FPG, TC, TG, high-density lipoprotein (HDL) cholesterol, low-density lipoprotein (LDL) cholesterol, and hemoglobin A1c (HbA1c) levels were measured using standardized laboratory methods. In addition, serum levels of N-terminal pro-B-type natriuretic peptide (NT-proBNP) were measured using an electrochemiluminescence immunoassay. All laboratory assessments were performed by the Department of Clinical Laboratory at the First Affiliated Hospital of Jinzhou Medical University. The estimated glomerular filtration rate (eGFR) was determined utilizing the Chronic Kidney Disease Epidemiology Collaboration (CKD–EPI) equation (13). Additionally, echocardiographic data were collected at enrollment and during the follow-up period. Left ventricular ejection fraction (LVEF) was measured using the biplane Simpson’s method. Patients with a history of peripheral vascular disorders, non-fatal myocardial infarction, stroke, atrial fibrillation, or hospitalization for HF at baseline were classified as having a history of cardiovascular diseases (CVDs).

In this study, the TyG-BMI index was calculated as follows: TyG-BMI = [ln (fasting TG (mg/dL) × FPG (mg/dL)/2)] × BMI (kg/m^2^) (8).

### The primary outcome

The primary outcome in this study was the diagnosis of HF using the ICD-10-CM code I50. Upon examining the patient’s medical records, we evaluate if they fulfill the following criteria (1): HF indicators include fatigue, dyspnea, and fluid retention; categorized as New York Heart Association functional class II, III, or IV; or Killip functional class II, III, or IV; (2) Assessment of plasma NT-proBNP concentrations: NT-proBNP ≥ 125 ng/L; (3) LVEF measured by echocardiography using the modified Simpson method, < 50%. The diagnosis of HF was confirmed if either condition (1) and condition (2) or condition (3) was met. We further validated the diagnosis by a group of cardiologists blinded to the study by chart review with a sensitivity of 92.6% and a specificity of 91.8%. In individuals with two or more instances of HF, the date of the initial episode was regarded as the endpoint. For patients who did not encounter any HF events throughout follow-up, the endpoint is designated as the date of death or December 31, 2024.

### Animal experiments

The Sprague-Dawley rats were used in this study. All animal experiments were supervised and approved by the Animal Care Committee at the Jinzhou Medical University. At 8 weeks of age, SD rats were randomly assigned to either a control group receiving a standard chow diet (SY10001F, Anhui Kuibu Shuyu Biotechnology Co., Ltd) or a treatment group receiving a high-fat/high-cholesterol (HFHC) diet (SY109C, Anhui Kuibu Shuyu Biotechnology Co., Ltd). SD rats were organized into groups and accommodated in cages within a facility that maintained a 12-hour light/dark cycle and a temperature range of 22-24°C, with unrestricted access to food and water. Fasting blood samples were collected at 24 weeks of age to determine the subsequent indicators. Levels of serum total cholesterol (TC), triglycerides (TG), creatine kinase (CK), and lactate dehydrogenase (LDH) were measured using an automatic analyzer (HITACHI 7600-120, Hitachi, Japan). A cardiac ultrasonography (Sono Vet Q70, Wuxi Xiangsheng Medical Technology Co., Ltd.) was conducted on rats at 24 weeks of age. The LVEF index was utilized to assess left ventricular systolic function.

### Statistical analysis

Normally distributed variables are expressed as mean ± standard deviation, whereas skewed variables are expressed as median and interquartile range. Categorical variables are represented by the frequency of cases and corresponding percentages. Inter-group comparisons utilized the student’s t-test for normally distributed variables, the Wilcoxon rank sum test for skewed variables, and the chi-square test for categorical variables. This study categorized the population into four groups according to the quartile levels of TyG-BMI, given the absence of a definitive clinical cut-off point for TyG-BMI. The incidence rate of HF was determined per 1000 person-years. A Kaplan-Meier curve with a log-rank test was employed to depict the cumulative incidence of HF across quartiles of TyG-BMI over the follow-up period. The Cox proportional hazards model was employed to estimate the hazard ratio (HR) and the 95% confidence interval (CI). The population attribution risk proportion of the lowest quartile of TyG-BMI on HF was calculated. The model was adjusted for variables including age, sex, blood pressure, LDL, HDL, HbA1c, smoking status, eGFR, use of antiplatelet or anticoagulant therapy, lipid-lowering medications, antihypertensive medications, glucose-lowering medications, and a history of CVD. Receiver operating characteristic (ROC) curve analysis was performed to evaluate the discriminative ability of TyG-BMI, BMI, and the TyG index for incident HF. Shapley Additive Explanations (SHAP) analysis was applied to interpret the contribution of each variable within the prediction model.

Statistical significance was set at *P* < 0.05. The data analysis for this study utilized the R statistical software package version 4.0.3.

## Results

### Baseline characteristics

The baseline characteristics are shown in **Table 1**. A total of 4116 patients (2941 men and 1175 women) were analyzed with a mean age of 63 years old. The median with an interquartile range of TyG-BMI levels at baseline was 208.5 (175 - 250). Patients were further divided into four groups according to TyG-BMI quartile levels: Q1 < 175, Q2: 175-208, Q3: 209-250, and Q4: ≥ 250. Patients exhibiting elevated TyG-BMI levels at baseline demonstrated increased HbA1c and were more likely to be utilizing antihypertensive and glucose-lowering pharmacotherapies. With the increase in TyG-BMI quartiles, clinical indicators of cardiac function showed a significant worsening trend. Baseline NT-proBNP levels significantly increased from a median of 124.5 pg/mL in Q1 to 205.7 pg/mL in Q4 (*P* for trend < 0.001). Concurrently, LVEF exhibited a downward trend, decreasing from 62.4 ± 5.8% in the Q1 group to 57.8 ± 8.4% in the Q4 group (*P* for trend < 0.05).

**Table 1:** Baseline characteristics of participants according to different TyG-BMI.

	TyG-BMI			
	<175	175~208	209~250	≥250
Participants (n)	1030	1030	1029	1027
Age (years)	68.6±12.0	67.6±11.3	65.1±10.8	61.0±10.8*
Male (%)	792 (76.82%)	758 (73.52%)	749 (72.51%)	642 (62.45%)*
Body mass index (kg/m^2^)	18.1±1.2	20.9±1.6	24.8±1.9	31.3±4.3*
Blood pressure (mmHg)				
Systolic	132±12	133±12	134±12	133±12
Diastolic	74±7	75±7	76±8	77±8
Total cholesterol (mg/dL)	162.3±38.6	163.4±40.3	165.6±37.4	165.1±35.2
Low-density lipoprotein cholesterol (mg/dL)	92.6±31.5	92.9±32.8	95.3±30.6	95.6±29.5
High-density lipoprotein cholesterol (mg/dL)	40.9±11.1	40.2±10.4	38.5±9.4	38.6±8.8*
Triglycerides (mg/dL)	147.3±86.1	157.9±112.7	164.7±94.1	159.9±98.6
Estimated GFR (mL/min/1.73 m^2^)	93.8±27.1	92.5±27.1	88.8±27.6	88.8±26.7
HbA1c (%)	7.3±1.5	7.4±1.6	7.5±1.6	7.6±1.5*
LVEF (%)	62.4 ± 5.8	61.2 ± 6.2	59.5 ± 7.1	57.8 ± 8.4*
NT-proBNP (pg/mL)	124.5 (82.1, 215.3)	142.8 (95.4, 258.6)	168.2 (110.5, 312.4)	205.7 (138.6, 425.8) ^*^
Current smoking (%)	107 (10.38%)	97 (9.41%)	87 (8.42%)	90 (8.75%)
Use of antihypertensive medications (%)	786 (76.24%)	798 (77.40%)	820 (79.38%)	831 (80.84%) *
Use of lipid-lowering medications (%)	647 (62.75%)	645 (62.56%)	686 (66.41%)	648 (63.04%)
Use of glucose-lowering medications (%)	721 (69.93%)	749 (72.65%)	803 (77.73%)	826 (80.35%) *
Use of antiplatelet or anticoagulant (%)	251 (24.35%)	214 (20.76%)	201 (19.46%)	218 (21.21%)
Incidence of Heart failure (%)	196 (19.01%)	215 (20.85%)	226 (21.88%)	267 (25.97%)
History of CVD	490 (47.53%)	487 (47.24%)	419 (40.56%)	378 (36.77%)

*p for trend <0.05.

### Association between TyG-BMI and incidence of HF

**Figure 2:**
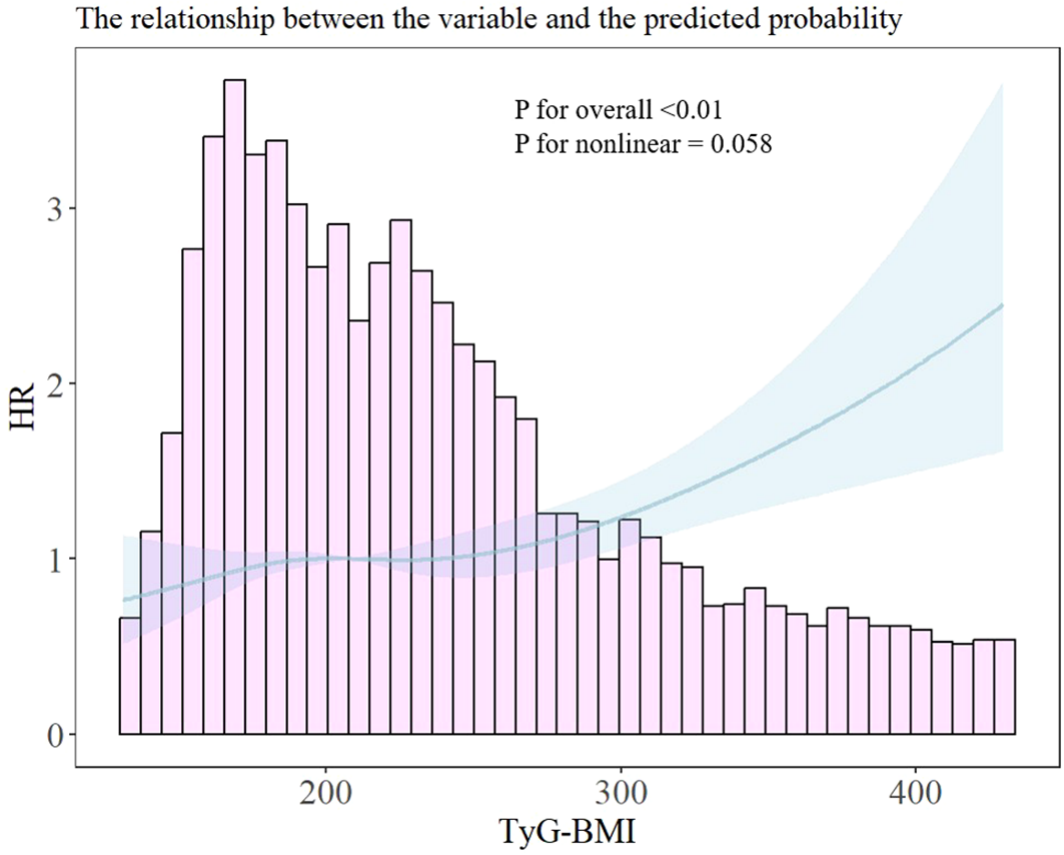
Restricted cubic spline analysis of the association between TyG-BMI and the risk of heart failure.

During a median follow-up of 3.01 years, 904 new cases of HF were reported. After adjusting for age and sex, the hazard ratios (HRs) (95% CI) of HF based on quartile of TyG-BMI levels at baseline [<175 (set as the reference), 175-208, 209-250, ≥ 250] were 1.00, 1.15 (0.90-1.42), 1.23 (1.02-1.38), and 1.50 (1.21-1.84), respectively, as shown in **Table 2**. After adjusting for multiple factors (age, sex, blood pressure, LDL, HDL, HbA1c, smoking status, eGFR, antiplatelet or anticoagulant, lipid-lowering medications, antihypertensive medications, glucose-lowering medications and history of CVD), the HRs (95% CI) for the Q2, Q3, and Q4 groups were 1.11(95% CI 0.89-1.45), 1.17 (95% CI 0.94-1.45), and 1.40 (95% CI 1.13-1.73), respectively (**Table 2**). When TyG-BMI was considered as a continuous variable, associated with a 13% increase in HF risk. The restricted cubic spline model (**Figure 2**) showed that an increase in TyG-BMI levels was positively associated with a high risk of HF.

**Table 2:** Hazard ratios of heart failure by different levels of TyG-BMI at baseline.

	TyG-BMI				As a continuous variable
	<175	175~208	209~250	≥250	
Heart failure					
No. of patients	1030	1030	1029	1027	
No. of cases	196	215	226	267	
Person-year	3101.2	3081.5	3113.8	2943.7	
Age- and sex-adjusted HR	1.00	1.15 (0.90-1.42)	1.23 (1.02-1.38) ^a^	1.50 (1.21-1.84) ^a^	1.25 (1.04-1.58)
aHR	1.00	1.11 (0.89-1.38)	1.17 (0.94-1.45)	1.40 (1.13-1.73) ^a^	1.13 (1.01-1.44)

Multivariable adjusted models included age, sex, blood pressure, LDL, HDL, HbA1c, smoking status, eGFR, antiplatelet or anticoagulant, lipid-lowering medications, antihypertensive medications, glucose-lowering medications and history of CVD.

^a^indicates that the hazard ratio is statistically significant (P < 0.05).

### Subgroup analyzes

We conducted subgroup analyzes to assess the consistency of the relationship between TyG-BMI and the occurrence of HF across significant clinical and demographic categories (**Table 1**). All analyzes were adjusted for the full set of covariates (age, sex, blood pressure, LDL, HDL, HbA1c, smoking status, eGFR, antiplatelet or anticoagulant use, lipid-lowering medications, antihypertensive medications, glucose-lowering medications, and history of CVD), and formal interaction testing was conducted to assess effect modification by each subgroup variable. A positive, dose-dependent association between TyG-BMI and HF risk was observed across all examined subgroups, consistent with the primary analysis in the overall population with type 2 diabetes. Formal interaction testing revealed no significant effect modification by any subgroup variable (all P for interaction >0.05), including age group (P = 0.824), sex (P = 0.756), BMI category (P = 0.691), HbA1c level (P = 0.912), and eGFR stage (P = 0.783). This confirms the generalizability of the positive TyG-BMI-HF association across the cohort with type 2 diabetes, with the strength and direction of the association unchanged by clinical or demographic characteristics.

### Predictive performance of TyG-BMI for incident HF

**Figure 3:**
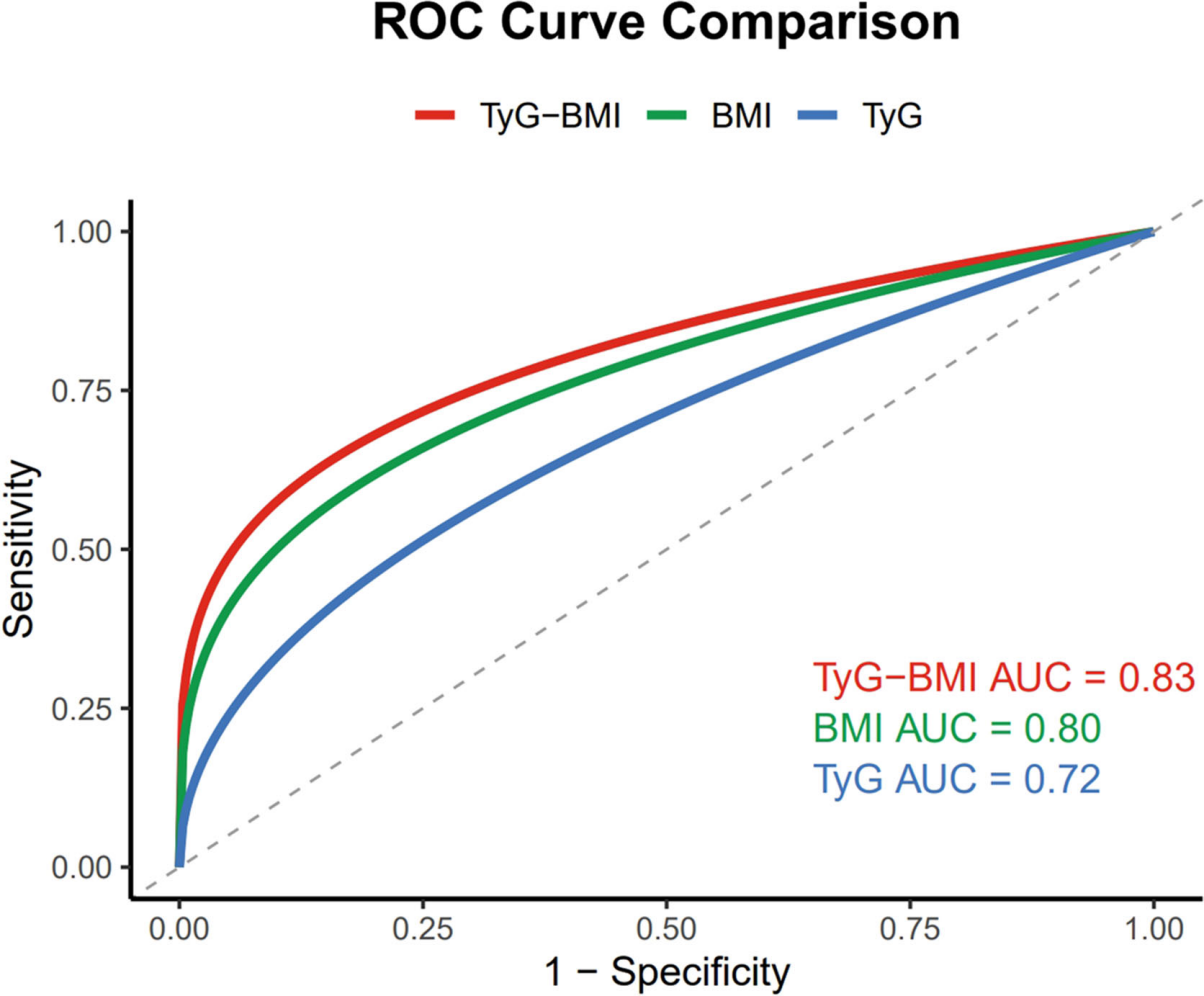
ROC curve comparison of TyG-BMI, BMI, and TyG index for predicting incident heart failure.

The ROC curve analysis was performed to evaluate the discriminative ability of TyG-BMI, BMI, and the TyG index for incident HF (**Figure 3**). The results showed that the TyG-BMI index exhibited robust predictive capability for incident HF, with an area under the curve (AUC) of 0.83, compared to 0.80 for BMI and 0.72 for the TyG index.

### Interpretability analysis using SHAP for HF risk prediction

**Figure 4:**
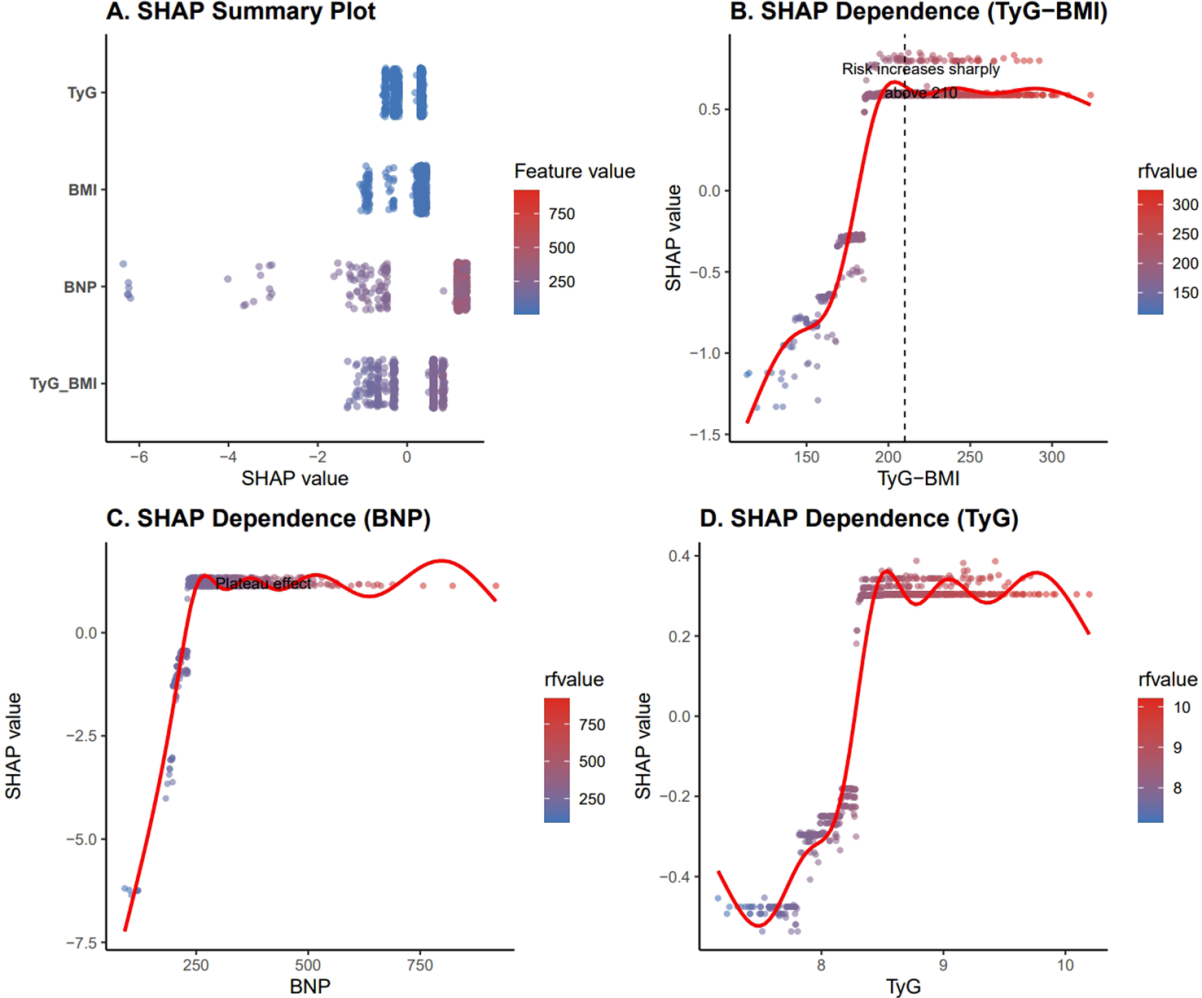
SHAP analysis for heart failure risk prediction.

In order to ascertain the extent to which individual variables contribute to the prediction of HF risk, SHAP analysis was performed (**Figure 4**). The SHAP summary plot demonstrated that TyG-BMI was the most influential feature in the model, followed by BNP, BMI, and the TyG index. Dependence plots further revealed a clear nonlinear relationship between TyG-BMI and HF risk, with a sharp increase in risk observed when TyG-BMI exceeded approximately 210, suggesting a potential threshold effect. BNP exhibited a rapid increase in SHAP values at lower levels followed by a plateau, indicating diminishing marginal effects at higher concentrations. In contrast, the TyG index showed a relatively modest and stable contribution across its range. Overall, these findings highlight the dominant role of TyG-BMI in driving model predictions and demonstrate its ability to capture complex nonlinear risk patterns for HF.

### Changes in blood glucose and lipid levels in rats with impaired cardiac function

**Figure 5:**
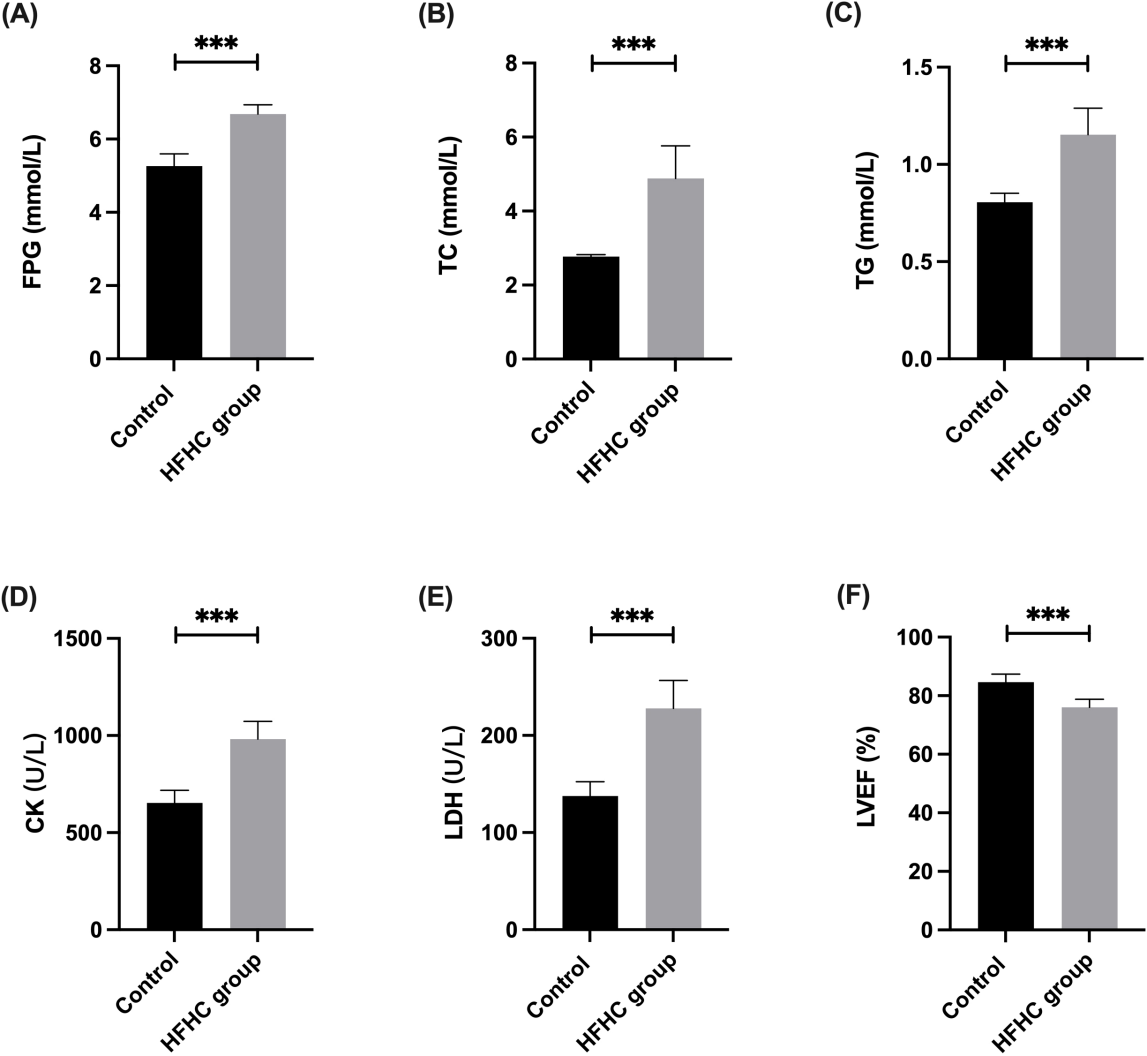
Changes in blood glucose and lipid levels in rats with impaired cardiac function.

Given the observed positive correlation between TyG-BMI and HF, we subsequently investigated changes in glucose and lipid metabolic parameters in the context of impaired cardiac function using SD rat. One cohort of rats was subjected to an HFHC diet, while the control cohort received a chow diet. The results indicated a significant elevation in blood glucose and lipid levels in the HFHC cohort (**Figures 5A–C**), accompanied by a notable elevation in serum levels of CK and LDH (**Figures 5D, E**). Cardiac ultrasound assessments revealed a significant decline in the LVEF in the HFHC group, indicating a severe impairment of cardiac function (**Figure 5F**).

## Discussion

This research examined the association between TyG-BMI levels and the onset of HF in Chinese individuals with type 2 diabetes. The findings indicated a positive correlation between baseline TyG-BMI levels and the risk of HF within the general population. The results of subgroup analysis also showed a dose-dependent positive correlation between TyG-BMI and the risk of HF. In comparison to BMI and TyG, TyG-BMI demonstrated a superior capacity for recognizing HF. In the context of impaired cardiac function, SD rats exhibited higher serum glucose and lipid levels. These experimental findings provide supportive evidence linking metabolic dysregulation to impaired cardiac function, although causal inference remains limited.

The triglyceride-glucose (TyG) index is widely recognized as a reliable and straightforward indicator for assessing IR (14). Numerous studies have established a strong correlation between TyG and the incidence, progression, and prognosis of HF (15–18). The TyG index can be integrated with several anthropometric parameters to generate several metrics, including TyG-waist circumference (WC), TyG-waist-to-height ratio (TyG-WtHR), and TyG-BMI. Er et al. (19) demonstrated that TyG-BMI is superior to other indicators, including visceral adiposity indicators, visceral adiposity index, lipid accumulation product, as well as TyG with other adiposity status in predicting IR. In recent years, several studies have investigated the association between TyG-BMI and HF in different populations. In a cross-sectional study involving 7472 individuals with diabetes or prediabetes, it was observed that participants in the highest tertile of baseline TyG-BMI exhibited a greater likelihood of developing HF (OR 2.645, 95% CI 1.529-4.576) compared to those in the lowest tertile. The restricted cubic spline analysis further indicated a significant dose-response relationship between TyG-BMI levels and HF (20). Additionally, a prospective cohort study involving 7335 individuals aged 60 and older revealed that, after an average follow-up period of 2.91 years, participants with a baseline TyG-BMI level below 142 or equal to or above 169 had a higher likelihood of developing HF, with HR and 95% CI of 1.17 (1.15-2.55) (21). Our findings were consistent in both the general population and subgroup analysis, demonstrating that a heightened TyG-BMI correlated with an elevated risk of HF.

In this study, we did not explore the relationship between TyG-BMI and HF-related adverse prognosis. However, when reading the research results of others, we discovered an interesting phenomenon, that is, there is no positive correlation between Tyg-BMI and HF-related cardiovascular mortality and all-cause mortality. For instance, an analysis of data from the National Health and Nutrition Examination Survey revealed that, after adjusting for various confounding factors, TyG-BMI levels in patients with HF were negatively correlated with all-cause mortality (OR = 0.69, 95% CI = 0.54-0.88) (22). Similarly, Chen et al. (23) found a negative correlation between TyG-BMI levels and the incidence of early new-onset HF in patients with acute ST-segment elevation myocardial infarction undergoing primary percutaneous coronary intervention. Additionally, Dou et al. (24) utilized the Medical Information Mart for Intensive Care database to evaluate the relationship between TyG-BMI levels and one-year all-cause mortality in patients with HF, revealing a negative correlation between elevated TyG-BMI levels and mortality risk (log-rank *P* = 0.003). We hypothesize that this may be associated with the incorporation of BMI in TyG-BMI. TyG-BMI is a composite indicator that integrates BMI, which in older demographics may signify nutritional status, physical reserve, or frailty rather than solely metabolic risk. The found inverse relationship in senior participants may be influenced by the BMI factor rather than insulin resistance itself.

An appropriate BMI range may be more critical for diminishing the likelihood of unfavorable outcomes. A prospective cohort study conducted in China examined data from 27,026 participants aged 80 years and above indicated that the risk of all-cause mortality, cardiovascular disease mortality, and non-cardiovascular disease mortality diminished with rising BMI. The most appropriate BMI range linked to decreased mortality was between 26.0 kg/m^2^ and 30.6 kg/m^2^ (25). We will further investigate the correlation between TyG-BMI and heart failure, along with associated long-term deleterious outcomes in the future.

The current study confirms that TyG-BMI is a clinically valuable prognostic biomarker for incident HF in patients with type 2 diabetes, with a consistent positive, dose-dependent association observed across all clinical and demographic subgroups. Furthermore, the recognition efficiency of TyG-BMI for HF surpasses that of BMI and TyG. This prognostic value stems from TyG-BMI’s unique ability to integrate three interrelated metabolic risk factors —TG, FPG and BMI, each of which independently contributes to cardiac dysfunction and HF development through distinct but synergistic pathological mechanisms. TG-induced myocardial lipotoxicity and steatosis, FPG-facilitated accumulation of advanced glycation end products and oxidative stress in cardiac tissue, and BMI-induced myocardial hemodynamic overload and fibrosis collectively contribute to the progression of diabetic cardiomyopathy and subsequent HF (26–30). Unlike single biomarkers, TyG-BMI captures the cumulative effect of these metabolic abnormalities, making it a more comprehensive tool for HF risk stratification.

There is currently no consensus on the relationship between TyG-BMI and HF. This study contributes data from a Chinese population with type 2 diabetes to the existing literature. This study has several limitations. This is a single-center retrospective cohort study undertaken in a Chinese community with type 2 diabetes, potentially restricting the generalizability of the findings to other ethnic groups, non-Asian populations, or individuals with T2DM from multi-center environments. Future multi-center, international cohort studies are needed to validate the TyG-BMI-HF association in diverse populations. TyG-BMI is a composite index that integrates BMI, a variable with multifaceted biological meaning, it may reflect nutritional status or physical frailty in other populations. Secondly, the study did not collect data on certain lifestyle and behavioral factors (e.g., dietary habits, physical activity, alcohol consumption) that may influence both TyG-BMI levels and HF risk. These factors may act as confounders, and their inclusion in future prospective studies would further strengthen the causal inference of the TyG-BMI-HF association. In addition, the animal experiment was exploratory in nature and was not designed to directly validate the clinical findings, which should be interpreted primarily based on the observational data. Finally, while the animal study confirmed elevated blood glucose and lipid levels in the context of impaired cardiac function, it did not directly investigate the relationship between TyG-BMI and cardiac dysfunction *in vivo*. Future preclinical studies are needed to establish a causal relationship between TyG-BMI and HF development and to elucidate the underlying molecular mechanisms.

## Conclusion

In conclusion, TyG-BMI levels exhibited a positive correlation with the risk of HF in populations with type 2 diabetes. In comparison to TyG and BMI, the TyG-BMI index exhibited superior predictive value for incident HF.

## Data Availability

The raw data supporting the conclusions of this article will be made available by the authors, without undue reservation.
