# Supplementary material for: Association between triglyceride glucose-body mass index and incident risk of heart failure among patients with type 2 diabetes: a real-world study
Source: Front Endocrinol (Lausanne). 2026 May 7;17:1798721. doi: 10.3389/fendo.2026.1798721 (PMC13189918; doi:10.3389/fendo.2026.1798721)
Supplement: Supplementary file 1 [file Table1.docx]

Supplementary Table S1. Subgroup analysis according to different baseline characteristics in association of baseline TyG-BMI with heart failure.

|  | TyG-BMI | | | | | | | *P* for interaction |
| --- | --- | --- | --- | --- | --- | --- | --- | --- |
|  | Q1  ≤0.47 | | Q2  0.48~0.64 | | Q3  0.65-0.88 | | Q4  ＞0.88 |  |
| Age, years old |  | |  | |  | |  | 0.824 |
| <65 | 1.00 | | 1.12 (0.89–1.40) | | 1.21 (1.00–1.46) | | 1.45 (1.18–1.78) |  |
| ≥65 | 1.00 | | 1.09 (0.88–1.35) | | 1.16 (0.95–1.42) | | 1.38 (1.11–1.72) |  |
| Sex |  | |  | |  | |  | 0.756 |
| Male | 1.00 | | 1.10 (0.88–1.37) | | 1.18 (0.96–1.45) | | 1.42 (1.14–1.77) |  |
| Female | 1.00 | | 1.13 (0.87–1.47) | | 1.20 (0.97–1.48) | | 1.39 (1.08–1.79) |  |
| Body mass index, kg/m^2^ |  | |  | |  | |  | 0.691 |
| <25 | 1.00 | | 1.15 (0.89–1.48) | | 1.23 (1.01–1.50) | | 1.48 (1.19–1.84) |  |
| ≥25 | 1.00 | | 1.08 (0.87–1.34) | | 1.15 (0.94–1.41) | | 1.37 (1.10–1.71) |  |
| HbA1c, % |  | |  | |  | |  | 0.912 |
| <7.0 | 1.00 | | 1.11 (0.88–1.39) | | 1.17 (0.95–1.44) | | 1.40 (1.13–1.73) |  |
| ≥7.0 | 1.00 | | 1.09 (0.88–1.35) | | 1.20 (0.98–1.46) | | 1.41 (1.14–1.74) |  |
| Estimated GFR, mL/min/1.73 m^2^ |  | |  | |  | |  | 0.783 |
| ≥90 | 1.00 | 1.13 (0.89–1.43) | | 1.19 (0.97–1.46) | | 1.44 (1.16–1.78) | |  |
| 60-89 | 1.00 | 1.10 (0.88–1.36) | | 1.17 (0.96–1.43) | | 1.39 (1.12–1.72) | |  |
| <60 | 1.00 | 1.07 (0.82–1.39) | | 1.22 (0.98–1.52) | | 1.46 (1.15–1.86) | |  |
| Current smoking |  |  | |  | |  | | 0.857 |
| No | 1.00 | 1.11 (0.89–1.38) | | 1.18 (0.96–1.45) | | 1.42 (1.15–1.75) | |  |
| Yes | 1.00 | 1.09 (0.81–1.46) | | 1.23 (0.95–1.59) | | 1.40 (1.08–1.82) | |  |
| Antiplatelet or anticoagulant |  |  | |  | |  | | 0.724 |
| No use | 1.00 | 1.12 (0.89–1.40) | | 1.19 (0.97–1.46) | | 1.43 (1.16–1.77) | |  |
| Use | 1.00 | 1.08 (0.86–1.35) | | 1.16 (0.94–1.43) | | 1.38 (1.11–1.72) | |  |
| Lipid-lowering medications |  |  | |  | |  | | 0.685 |
| No use | 1.00 | 1.14 (0.88–1.47) | | 1.22 (0.99–1.50) | | 1.47 (1.18–1.83) | |  |
| Use | 1.00 | 1.09 (0.88–1.35) | | 1.17 (0.95–1.44) | | 1.39 (1.12–1.72) | |  |
| Antihypertensive medications^‡^ |  |  | |  | |  | | 0.791 |
| No use | 1.00 | 1.10 (0.84–1.44) | | 1.18 (0.93–1.49) | | 1.45 (1.13–1.86) | |  |
| Use | 1.00 | 1.11 (0.89–1.38) | | 1.18 (0.96–1.45) | | 1.41 (1.14–1.74) | |  |
| Glucose-lowering medications |  |  | |  | |  | | 0.715 |
| No use | 1.00 | 1.13 (0.88–1.45) | | 1.21 (0.98–1.49) | | 1.46 (1.17–1.82) | |  |
| Use | 1.00 | 1.09 (0.88–1.35) | | 1.17 (0.95–1.44) | | 1.39 (1.12–1.72) | |  |

Data are hazard ratios (95% confidence intervals) unless otherwise indicated. Multivariable adjusted models included age, sex, blood pressure, LDL, HDL, HbA1c, smoking status, eGFR, antiplatelet or anticoagulant, lipid-lowering medications, antihypertensive medications, glucose-lowering medications and history of CVD

Supplementary Table S2: Sensitivity Analysis: TyG Index Alone and Incident HF (BMI as an Independent Covariate)

| **TyG Index Quartile** | **HR (95% CI)** | ***P*-value** |
| --- | --- | --- |
| Q1 (Ref) | 1.00 | - |
| Q2 | 1.08 (0.86-1.35) | 0.512 |
| Q3 | 1.12 (0.90-1.40) | 0.317 |
| Q4 | 1.25 (1.01-1.54) | **0.041** |

Multivariable adjusted models included age, sex, BMI, blood pressure, LDL, HDL, HbA1c, smoking status, eGFR, antiplatelet or anticoagulant, lipid-lowering medications, antihypertensive medications, glucose-lowering medications and history of CVD

Supplementary Table S3. Independent Contributions of TG, FPG, and BMI to Incident HF

| **Variable** | **HR (95% CI)** | ***P*-value** |
| --- | --- | --- |
| Triglycerides (TG) | 1.001 (0.999–1.003) | 0.215 |
| Fasting plasma glucose (FPG) | 1.002 (0.998–1.006) | 0.307 |
| Body mass index (BMI) | **1.04 (1.01–1.07)** | **0.008** |

Supplementary Table 4 Multivariable-adjusted Hazard Ratios (HRs) for incident Heart Failure associated with TyG index after further adjustment for BMI.

| **Model** | **Variable** | **HR (95% CI)** | ***P*-value** |
| --- | --- | --- | --- |
| Model A (TyG only) | TyG index (per SD increase) | 1.28 (1.12–1.45) | <0.001 |
| Model B (BMI only) | BMI (per SD increase) | 1.15 (1.04–1.27) | 0.006 |
| Model C (TyG + BMI) | TyG index (per SD increase) | 1.22 (1.08–1.38) | 0.002 |
|  | BMI (per SD increase) | 1.09 (0.98–1.21) | 0.112 |

Model A includes the TyG index only.
Model B includes BMI only.
Model C includes both the TyG index and BMI simultaneously.
Multivariable adjusted models included age, sex, BMI, blood pressure, LDL, HDL, HbA1c, smoking status, eGFR, antiplatelet or anticoagulant, lipid-lowering medications, antihypertensive medications, glucose-lowering medications and history of CVD
